# Hepatic hemangiomas with arterioportal shunt complicated a congestive heart failure: a case report

**DOI:** 10.11604/pamj.2023.44.181.39820

**Published:** 2023-04-18

**Authors:** Sara Aminou, Nadia Mebrouk, Loubna Chtouki, Abdelali Bentahila

**Affiliations:** 1 Department of Pediatric Cardiology, Mohammed V University, Children's Hospital of Rabat, Rabat, Morocco

**Keywords:** Hepatic hemangioma, arteriovenous shunt, propranolol, case report

## Abstract

Diffuse miliary haemangiomatosis is a rare condition representing 2.5-3% of cases of cutaneous infantile haemangiomas. It is an efflorescence of five or more infantile cutaneous haemangiomas associated with visceral involvement, most commonly liver involvement. The severity is mainly related to the risk of congestive heart failure. These vascular anomalies are characterised by their clinical, evolutionary and structural polymorphism. The prognosis, whether aesthetic, psychological, functional or vital, is very heterogeneous, which conditions their frequently multidisciplinary management. The objective of this work is to report a complicated form of miliary hemangiomatosis illustrating clinical, radiological and biological particularities.

## Introduction

Hemangiomatosis miliaris is the efflorescence of 5 or 6, and up to several hundred, infantile hemangiomas where skin lesions are associated with visceral involvement. Haemangiomas may be present at birth, but more often they appear in the first few weeks of life and up to a fairly late age of 12-18 months [1]. Diagnosis is clinical, and abdominal ultrasound is required to look for hepatic haemangiomas. These can be asymptomatic, but can also be complicated. We report the observation of a 3-month-old infant who had multiple cutaneous haemangiomas of a few millimeters in size since 20 days of life and the hepatic ultrasound showed hepatic haemangiomas associated with an arteriovenous shunt.

## Patient and observation

**Patient information:** infant aged 3 months, second of two siblings having a mother of 27 years old, no particular pathological history, blood group B+. From a non-consanguineous marriage. Pregnancy poorly monitored, carried out at 35 South African according to Dubovitz, negative infectious anamnesis. Delivery by medical vaginal route, birth weight=1600g, Apgar not specified, mixed breastfeeding, only vaccinated against viral hepatitis B. Hospitalization at 20 days of life for hemangiomatosis miliaris.

**Figure 1:**
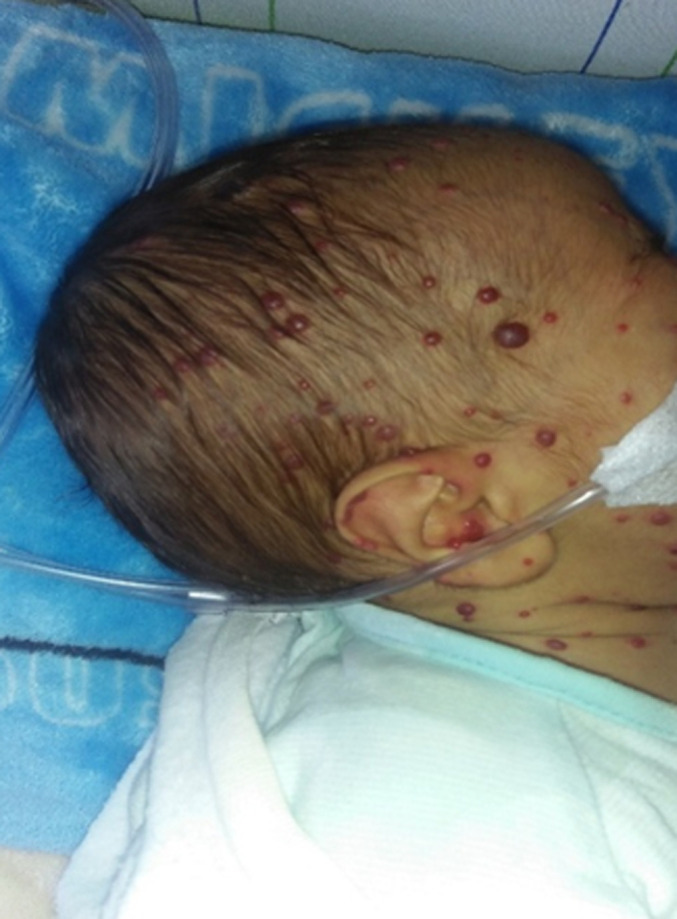
generalized cutaneous hemangioma

**Figure 2:**
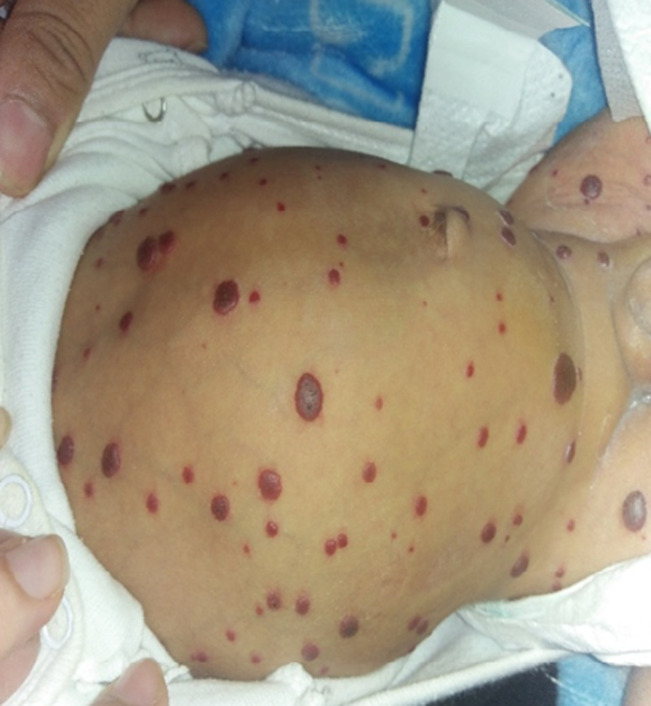
distended abdomen with venous circulation

**Clinical findings:** icteric infant, apyretic, weight=4900g, height=50cm, cerebral palsy (CP)=35cm, BP=102/64mmgh, HR=150bpm, FR=66c/min, cardiac resynchronization therapy (CRT) <3sec, SaO2=88% on room air. Signs of respiratory struggle such as supra sternal subcostal and intercostal pulling. Cardiac murmur and diffuse snoring on cardiorespiratory auscultation. Anterior fontanel was normotensive. There were small red tumors, with sharp borders, a mamelinated and tense surface, a firm consistency, varying in size from a few millimeters to 2 centimeters (Figure 1). These lesions were located all over the body including the soles, palms and lips. Distended abdomen with collateral venous circulation, liver and spleen difficult to palpate (Figure 2). Well-differentiated male external genitalia with left hydrocele.

**Figure 3:**
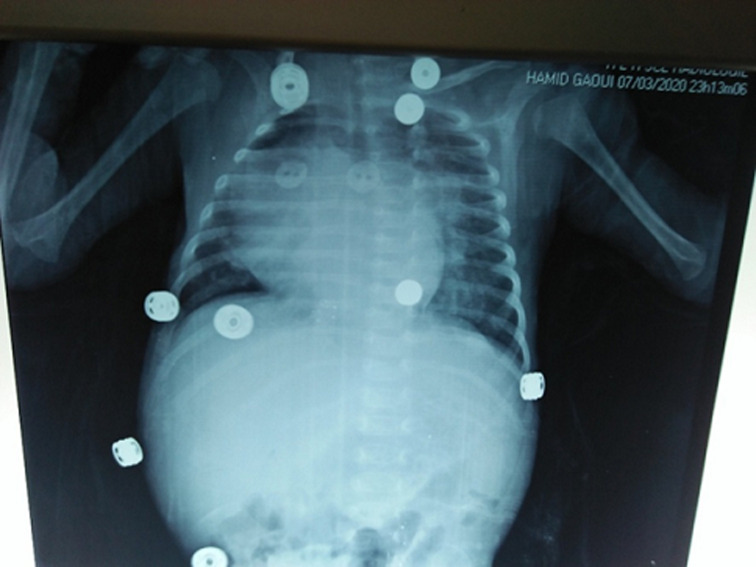
frontal thoracoabdominal radiograph: cardiomegaly

**Figure 4:**
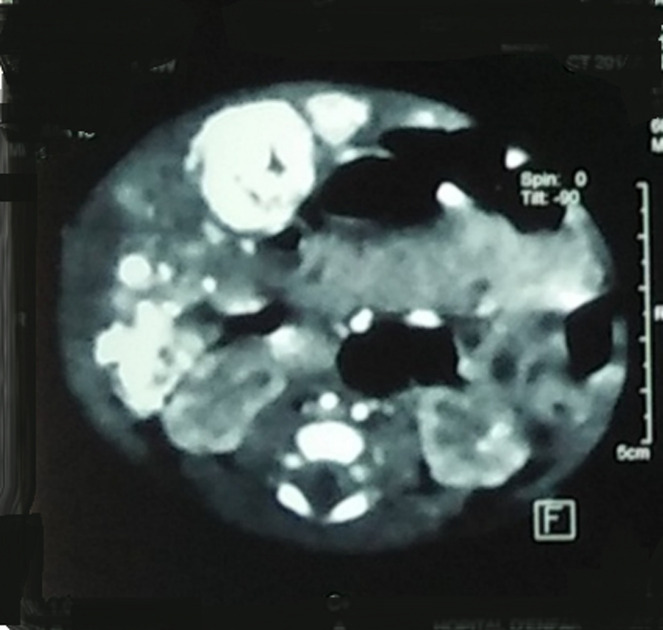
abdominal computed tomography cross-section: hepatic angiomas

**Figure 5:**
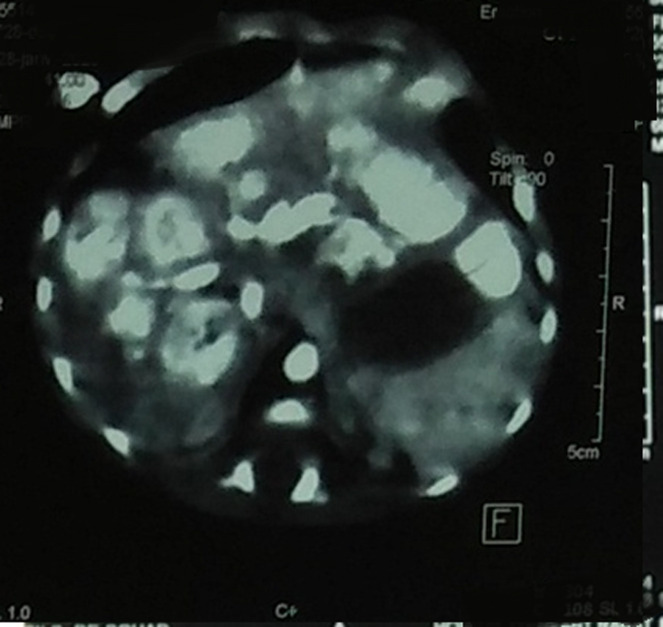
abdominal computed tomography cross-section: arteriovenous shunts

**Diagnostic approach:** the biological work-up showed hepatic cytolysis with aspartate aminotransferase (ASAT)=564 IU/L (16xN), alanine amino transferase (ALAT)=175 IU/L (7xN), PAL=1159 IU/L, gamma-glutamyl transferase (GGT) =63U/L, BT=217 mg/l, normal renal function, infection work-up was negative, PT=60%, partial thromboplastin time test (APTT)=1.3, blood grouping A+, Hb=11g/dl, white blood cell (WBC)=6460/mm^3^, patient location tracking and query (PLQ)=285,000/mm^3^. Chest X-ray showed cardiomegaly (Figure 3). On cardiac echo, there was grade III mitral insufficiency, moderate tricuspid insufficiency, and tiny trabeculated inferior vena cava (IVC). The echo-abdominal scan showed an enlarged liver with multiple hypo-echoic, well-limited, hyper-vascularized lesions on Doppler, the largest of which was located in segment II and measured 21x21cm; dilatation of the portal branches of the hepatic veins and the hepatic artery; individualization of arteriovenous and veno-venous shunts. Abdominal and renal computed tomography (CT) scans showed an enlarged liver (7cm) with multiple hypodense lesions in spontaneous contrast, intensely enhanced in the periphery at arterial time with centripetal filling at portal time; the most voluminous were located and measured 22x24 mm in segment V and 27x18 mm in segment VIII (Figure 4); early enhancement of VSH and portal branches at arterial time testifying to the presence of arteriovenous fistulas; individualization of shunts between segmental portal branches and VSH (Figure 5); left pyloric ectasia without individualize obstruction; pelvic intraperitoneal effusion slide. The radiological appearance was suggestive of diffuse hepatic angiomas with intrahepatic, arteriovenous (AV) and venovenous shunts. Cerebral CT was without abnormalities. Ultrascrotal examination showed a left inguino-scrotal hernia with signs of pain.

**Therapeutic intervention and follow-up:** the infant had received: hydrocortisone hemisuccinate (HSHC) corticotherapy =5 mg/kg/6h, propranolol= 2 mg/kg/dr, furosemide injection= 1 mg/kg/8h and vitamin K. The evolution was marked by the installation of rectorrhagia of small abundance, then aggravation of the respiratory distress followed by cardio-respiratory arrest.

**Patient perspective:** infant died at the stage of hepatocellular failure, complicated by a hemorrhagic syndrome.

**Informed consent:** written informed consent was obtained from the patient's parents.

## Discussion

Haemangioma is the most common benign tumour, affecting 10-12% of infants, and is present in 30% of premature infants weighing less than 1800g [1]. It is a tumour formed by a transient hyperplastic proliferation of angioforming mesenchyme, a cluster of endothelial cells fed and drained by neovessels. The initial stimulus for its growth is also unknown, but recent studies support a role for stress and prenatal anoxia [1]. According to Hidano, 75% of haemangiomas appear after the third week of life [2]. However, the severe visceral form is very rare, accounting for 2.5-3% of cases of cutaneous infantile haemangiomas [2]. Hepatic haemangiomas account for almost 90% of hepatic vascular anomalies in children [2]. The study by Kassarjian *et al*. found, in a series of 55 hepatic haemangiomas in children, 40% solitary and 60% multifocal [2]. Infantile hepatic haemangiomas can lead to serious complications that can be life-threatening: congestive heart failure due to the association with large arterio-portal shunts (this was the case for our patient); Kasabach-Merrit syndrome (coagulopathy due to intra-lesional platelet sequestration); severe hypothyroidism; anaemia and haemoperitoneum [3]. In 1914, Falkowski first reported the occurrence of heart failure in an infant with miliary haemangiomatosis [3]. The association between a haemangioma and an arterio-portal shunt (AP) is reported with an incidence of up to 26% [3]. This association is significantly more frequent with haemangiomas with rapid enhancement kinetics, i.e. capillary haemangiomas. The presence of transient peri-lesional enhancement is significatively more frequent with a small hemangioma (< 2cm) than with a hepatocellular carcinoma of the same size [4]. On colour Doppler, it is possible to visualise the afferent arterial flux and the efferent portal flux. After injection, this shunt results in early opacification of the adjacent portal branches. On ultrasound, these hemangiomas are most often hypoechoic and homogeneous. On colour Doppler, it is possible to visualise an intra-lesional flux [4]. On CT scan, these small haemangiomas appear discretely hypodense without injection, similar in density to the aorta, but may also be isodense and therefore sometimes undetectable [5].

Early, intense, homogeneous contrast enhancement is observed, similar to the aortic enhancement in the arterial phase. In the late stages, this enhancement follows that of the aorta, which makes it possible in particular to differentiate haemangioma from hepatocellular carcinoma (HCC) and certain hypervascular metastases [6]. On Magnetic resonance imaging (MRI), these small lesions also show a frank and homogeneous T2 hyper signal and contrast kinetics similar to those seen on CT with uniform and rapid contrast uptake [6]. Studies have reported significant elevation of alpha fetoprotein levels associated with hepatic haemangiomas [7]. The gastrointestinal tract is the second most common location and the risk is mainly haemorrhagic, sometimes in the form of occult bleeding; this was the case in our patient who presented with rectal bleeding. Airway involvement can be seen in haemangiomatosis and can lead to life-threatening obstruction. Involvement of the central nervous system is exceptional, and can result in headaches, convulsions, and sometimes hydrocephalus with intracranial hypertension. Ocular involvement is also possible with a risk of anisometropic amblyopia, glaucoma or retinal haemorrhage [6]. Haemangiomatosis can be confused with almost all liver tumours: hepatoblastoma, metastatic neuroblastoma, angiosarcoma, mesenchymal hamartoma, metastases, neonatal myelocytic leukaemia, as well as venous or arterial vascular malformations [8]. Infantile myofibromatosis may take the form of miliary haemangiomatosis with visceral involvement. Neonatal Langerhans histiocytosis of the Hashimoto-Pritzker type may have a Pseudoangiomatous appearance [8]. Another differential diagnosis to consider is blueberry muffin baby, characterised by multiple dark blue nodules and classically associated with fetal infections (Torch syndrome), or leukaemia [8]. The efficacy of medical treatment alone in diffuse miliary haemangiomatosis with liver involvement has been highlighted by many authors [4]. Several drugs have been proposed alone or in combination: propanolol, systemic corticosteroids, vincristine and interferon. A retrospective multicentre study showed that propranolol treatment was clinically more effective, with an improvement in the lesion of 75% or more compared to prednisone, and was better tolerated than systemic corticosteroids [9]. Propranolol should be given as early as possible, at a minimum of one month of age and during the proliferation phase of the haemangioma. It can be initiated gradually with a starting dose of 1mg/kg/d, increased in steps to an effective dose of 2-3 mg/kg/d on an outpatient basis and in some cases under monitoring [10]. It is advisable to treat until the end of the growth phase of the infantile haemangioma and to gradually reduce the dose when treatment is stopped. In the special case of hepatic haemangiomas, embolisation of atrioventricular (AV) or arterioportal (AP) shunts by interventional radiology may be used in cases of heart failure refractory to medical treatment [10].

## Conclusion

There is great clinical and prognostic variability within miliary haemangiomatosis, ranging from asymptomatic multifocal hepatic haemangiomatosis to dreadful digestive or neurological forms. Hepatic haemangiomatosis warrants close cardiovascular monitoring. Propranolol is used as a first-line treatment before general corticosteroid therapy in these newborns. Miliary haemangiomatosis has a poor prognosis and the mortality rate can reach 90%.
